# Medical Opinion and Movement

**Published:** 1910-10-22

**Authors:** 


### Medical Opinion and Movement.

### Acute Post-operative Dilatation of Stomach.

Dr. Lardennois has propounded the somewhat novel theory in regard to the pathology of acute dilatation of the stomach following operation that it is due, at any rate in a large number of cases, to the repeated and continual swallowing of air and saliva by the patient. His line of argument is that many of these patients are nervous and dyspeptic, suffering from thirst and dryness of the throat irritated by the anæsthetic, and sometimes predisposed to aerophagy. They multiply mechanically the movements of deglutition, partly from ennui and partly to swallow their saliva and assuage their thirst. In this way they accumulate atmospheric air in their stomach, already probably dilated and paralysed. The over-distended stomach compresses the coils of intestine, and then results acute post-operative occlusion of the duodenum. The author points out the similarity between the symptoms of these post-operative conditions and the serious attacks of collapse that have been observed by physicians in certain aerophagists. This repeated and more or less unconscious deglutition may certainly be frequently observed in patients after operation, and, according to the author, the introduction of a tube into the stomach is frequently followed by the evacuation of a large volume of gas, and such an evacuation is usually accompanied, as in the case of aerophagists, by a complete subsidence of the symptoms.

### Pericardial Irritation.

Dr. Hirtler has carried out a series of experimental researches on animals by which he has been able to demonstrate that mechanical or electric excitation of the pericardium produces a cardiac arrythmia, but that this phenomenon does not appear if the irritability of the pericardium is annihilated by the application of cocaine. He is of opinion that these observations explain the arrythmia that frequently accompanies the onset of a pericarditis, and also other cardiac symptoms of a serious nature that may be manifested in the course of such an attack, although they have generally been attributed to involvement of the myocardium in the morbid process. In further confirmation of this idea he cites a case of a wound inflicted in the precordial region by a revolver shot. The gravity of the cardiac symptoms led to the supposition of a wound of the heart. On operation, however, the heart was found to be intact, but there was a wound of the pericardium, and the pericardial sac contained air and blood. The serious symptoms disappeared as soon as the pericardial cavity was opened up. In other surgical interventions on the heart great irregularity of the cardiac activity has been observed, especially during suturing of the pericardium, and this agrees with Dr. Hirtler's observations on animals. The practical outcome of these observations is that the author recommends that in all cases of operative interference with the pericardium it should be first cocainised with a 10 per cent. solution so as to inhibit pericardial irritation, and so prevent the occurrence of cardiac arrythmia. At the end of the operation the cocaine can be got rid of by lavage with a physiological salt solution.

### Powdered Silver Nitrate for Open Wounds.

Dr. Max Barnet, of Berlin, recommends the use of powdered silver nitrate as a means of exciting the proliferation of granulations and the regeneration of epidermis over open wounds and ulcers. As an excipient he uses fullers' earth. The latter should be sterilised by heating to 100° or 150° C., which increases also its hydroscopic properties. The mixture should be used in the proportion of 1 part of silver nitrate to 99 parts of the earth. It is dusted on the raw surface, care being taken not to let it extend over the parts already healed over, and renewed every second, third, or fourth day according to the amount of secretion and reaction of the tissues. When the wound is well on the way to epidermisation it is recommended to interrupt the treatment from time to time and apply a simple aseptic dressing. The author has employed the treatment in a large number of cases, and recommends it especially for burns and for the healing of wounds following furuncles and other infective processes of the skin.

### Rice Diet for Skin Affections.

The close connection between diet and various skin affections, such as urticaria and eczema is a well recognised fact. Certain foods are known to be the direct cause in susceptible people of outbreaks of urticaria; and in the case of eczema, although the causal connection may be not so close, it is undoubtedly affected by diet, and a restricted diet always forms part of a rational treatment. Dr. L. D. Bulkley, of the New York Skin and Cancer Hospital, has conceived the idea of putting such patients on a diet consisting chiefly of rice. He first tried this four years ago on a patient suffering from a severe attack of polymorphous erythema with bullæ. The patient had had two previous attacks, and this one followed a heavy meal with champagne. A laxative and an alkaline lotion were prescribed, and a diet of boiled rice, bread and butter, and water. The attack subsided in five days, and the patient was surprised at the rapidity of the cure in comparison with the previous attacks. Since then the author has prescribed the diet in all manner of acute and subacute affections of the skin, and is much impressed with the good effects. It has shown excellent results in cases of general eczema, which are frequently so obstinate. The author recommends the rigid diet for about five days, and then a light mixed meal may be taken midday, the rice and bread and butter being continued night and morning. The rice must be cooked carefully in a little water (never in milk) and left on the fire uncovered till it becomes more or less dry and flaky.

### Optic Neuritis in Chlorosis.

A writer in the *British Medical Journal* committed himself last July to the statement that optic neuritis in chlorosis is probably far from uncommon. Dr. C. O. Hawthorne objects to this hypothesis, and states that he can recall but six cases of the association, though he had for many years made a point of examining the fundus of every patient who comes under his care. The point is of interest, partly because the exact incidence of optic neuritis in chlorotics is still a matter of doubt, partly because the nature of chlorosis is still obscure, and partly because it is not easy to explain with confidence how the two conditions are related. An American author, Dr. W. C. Posey, quoted in the *Ophthalmoscope*, records a typical case of double neuro-retinitis in a girl of twenty-one suffering from a not very severe form of chlorosis. It would appear that the literature to date includes less than thirty cases, though it may be suspected, as the *British Medical Journal* hinted, that the majority go unreported. Dr. Hawthorne ascribes both this lesion and paralysis of the external rectus (which sometimes complicates chlorosis) to intracranial thrombosis; Stölbing puts it down to vascular sclerosis of the cavernous sinus. Others regard this optic neuritis as due to toxæmia, a not improbable factor in any case of chlorosis. Cases of simple chlorosis are so seldom admitted to hospital wards that it is reasonable to believe that many others are missed for lack of ophthalmoscopic examination, especially as vision is not necessarily much affected.

### Auto-Laparotomy in Pregnancy.

It seems almost incredible that a woman should perform Cæsarean section upon herself and yet survive; but the fact is well attested. Two such cases have lately been published by Kurt von Sury. The details are given in the *Medical Review*. A girl of nineteen, who had concealed her pregnancy, tried to commit suicide by ripping the abdomen with a razor. The blade not only pierced the parietes, but the posterior surface of the uterus as well. The infant was found drowned in a tub, where it had fallen by accident. The abdominal and uterine wounds were sutured and the patient recovered. In the other case a woman of thirty-eight got tired by a tedious labour and stabbed herself with a penknife in the hope of stopping the pains. The uterus was wounded in four places, but the child was undelivered. Cæsarean section was performed, but the patient died later of septicæmia. Still more extraordinary is the case reported by Baliva and Serpieri. A peasant girl made an abdominal incision upon herself and opened the uterus. She extracted the child after severing the head from the body. The intestine prolapsed, but she replaced it, bandaged her abdomen, and actually went about her work for several hours before her condition was discovered. She eventually got quite well. Yet another successful auto-laparotomy is recorded by von Guggenbus on the part of a woman who got uterine tetanus. Feeling herself *in extremis* she performed laparotomy most methodically, dividing the tissues layer by layer. She opened the uterus, extracted child and placenta, and also cut the umbilical cord. In another case of the same sort published a few years ago from one of the Balkan States the patient even sewed herself up with needle and thread after doing Cæsarean section upon herself.

### Alcohol as a Dressing for Wounds.

Even before the general recognition of the bacterial origin of sepsis Dr. H. T. Bahnson was using alcoholic solutions for dressing wounds; and after forty years, during which he has tried every antiseptic in turn, he returns to his first love. For this purpose he uses as a rule an equal-parts mixture, but when he thinks it necessary he employs alcohol pure. After operation the first dressing is soaked in this fluid, and is moistened later whenever the dressings get stiff. It is also recommended as an efficient first dressing for burns; and boils, carbuncles, and whitlows are said to be absorbed or limited if kept moistened with it. In suppurating abdominal cases drains are removed on the third or fourth day, and irrigation is abandoned for gentle mopping out of the cavity with dilute alcohol. For diffuse cellulitis of the limbs he has given up multiple incisions. Instead he advises, first, absolute rest, including scrupulous avoidance of all manipulation, palpation, etc.; and, second, the enveloping of the whole limb in a loose voluminous dressing of gauze and absorbent cotton, kept constantly wet with a fluid consisting of one part of alcohol and four to six parts of saturated boric acid solution. This dressing is covered with some impervious material to retain warmth and check evaporation. The warm fluid is poured on as often as is necessary to keep the whole dressing wet—not merely damp—and the dressing is renewed in forty-eight hours. The details of the method, with some illustrative cases, are given in Dr. Bahnson's original paper in the *International Journal of Surgery*.

### The Thymus Gland.

According to the *Berlin Klin. Woch.*, C. Hart and O. Nordman find that the extirpation of the thymus gland in young dogs results in the death of the animals within twelve months. The operation is followed by no acute symptoms, nor do rickets occur if the animals be supplied with plenty of air, light, and food rich in lime salts. In the first months a certain degree of retardation in growth is to be noticed, but no marked skeletal changes are to be found as compared with control animals. The general condition of nutrition is a little below standard, despite the avidity wherewith meals are devoured. The sexual organs retain their infantile characteristics, pregnancy and the sexual instincts being absent. Muscular development is poor, and fatigue comes on readily. No obvious cause of death is to be found post-mortem. There is noticed, however, a dilatation of the cardiac ventricles with thinning of their muscular walls. Nothing definitely abnormal is seen in the nervous system or the other ductless glands. The authors also experimented with a view to determining the effects on the system of excessive amounts of thymus gland. Portions of the organ were implanted subcutaneously in healthy dogs. At first moderate symptoms of intoxication were met with, but these quickly subsided. None of the animals died, so that no post-mortem observations were taken. The excessive tissue was in all probability quickly removed by absorption. The authors therefore conclude that the thymus is an organ which is essential to early life, and that it has a direct action on food absorption and the regulation of the circulation. It has in all probability some influence in addition upon the increase in resistance of the organism to bacterial infection. Partial extirpation has no ill-effects, while implantation of excess of the gland causes only temporary intoxication.

### Economy in Hypodermic Needles.

THE *Medical Record* draws attention to the carelessness of members of the profession in throwing away hypodermic needles which have become occluded owing to the deposition of material derived from the fluid used for injecting. All that is necessary to be done in order to restore the brightness of the external surface of such needles, as well as to cleanse them internally by dissolving out the precipitated material, is to boil them for a period of ten minutes in a solution of sodium bicarbonate in water. The economy of such a procedure is obvious, for the sum total of hypodermic needles thrown away by practitioners must amount in the course of a year to many thousands, which, in view of the cost of such articles, must represent a large sum of money.

### Hæmophilia of the New-born.

IN the *American Journal for the Medical Sciences* J. E. Welch describes a new treatment for hæmophilia neonatorum. Characterised by general diffuse hæmorrhage, apparently due to some change in the chemical composition of the blood whereby clotting is prevented, the disease responds badly to treatment by the administration of hæmostatics; saline infusion affords but temporary benefit, and the injection of animal blood or serum makes matters worse by the resulting hæmolysis. Carrel's treatment by anastomosis of a vein of the infant with an artery of a healthy adult has given good results, but its application is not easy, as a willing "blood-giver," is not always at hand, and moreover it needs delicate technique. The author of the present paper proposes to overcome this difficulty by drawing off the donor's blood into a sterile flask, allowing the serum to separate, and then injecting the latter in separate doses of 10 c.c. at a time subcutaneously. In severe cases two-hourly injections are given. He has treated eight cases by the method, all of which recovered. Out of eighteen cases treated by other methods in the New York Lying-in Hospital, seventeen died. He is unable to give an explanation of the way in which the serum acts in overcoming the hæmorrhagic tendency, but his success with this method in a disease which is usually fatal makes it worthy of being given a trial.

### Irritation of Skin from Mercurial Lotion.

AN extraordinary case of susceptibility to poisoning by mercurial lotion is reported in *Le Progrès Médical* by Drs. Tissier and Corpechot. The patient, a healthy woman of thirty-two, was admitted to hospital for her sixth pregnancy. She gave a history of having suffered at her previous confinements from an eruption of the lower abdomen, inner surface of the thighs, and round the anus, which she attributed to the use of corrosive sublimate for cleansing the parts during the puerperium. With the exception of a rash which covered the whole of her body at a time when her first child died of meningitis at the age of nine months, no history of cutaneous eruptions other than those connected with her lying-in could be obtained. In consequence of her statement, care was taken to avoid using mercurial preparations for sterilisation purposes during her lying-in. After the child was born, however, and before the placenta had been removed, the midwife sterilised her hands in a solution of 1 in 1,000 oxycyanide of mercury. She then expressed the placenta. The same evening the patient complained of a smarting in the region of the umbilicus, and a patch of erythema was noticed corresponding in position to the place where the midwife's hand had rested when applied to the fundus uteri. Next day this patch was even more defined, the affected skin being red and slightly raised above the surrounding level, minute vesicles containing fluid being scattered over it. This fluid was at first colourless, but gradually became turbid and yellow. A sensation of itching was present. The erythema faded gradually after the third day. At no time were there any general symptoms, nor were there any signs of an eruption elsewhere on the body.

### Alum Baths in Typhoid Fever.

ACCORDING to an article in the *Journal of the American Medical Association* by Dr. A. R. Boggs, the use of alum baths in typhoid fever obviates to a large extent the incidence of skin affections. One pound of powdered alum is dissolved in a little hot water and added to the water in the bath. In the average bath of 450 to 500 litres this makes approximately a 1 in 1,000 solution. The cost works out at about 2d. a bath. The patient is bathed in the ordinary way, and experiences no unpleasant results from the drug in solution. During desquamation a slight increase of the amount of skin thrown off is noticed. Of course, the treatment is not intended to replace that instituted ordinarily for the preservation of the skin in a good condition. Strict care of the skin must be adhered to. The author claims that with these baths and proper attention to the skin of the patient the incidence of skin complications is reduced by something like 50 per cent.

